# METTL3 Potentiates M2 Macrophage‐Driven MMT to Aggravate Renal Allograft Fibrosis via the TGF‐β1/Smad3 Pathway

**DOI:** 10.1002/advs.202412123

**Published:** 2025-01-27

**Authors:** Qinfan Yao, Xiaoxiao Zheng, Xinyi Zhang, Yucheng Wang, Qin Zhou, Junhao Lv, Li Zheng, Jiahua Lan, Wei Chen, Jianghua Chen, Dajin Chen

**Affiliations:** ^1^ Kidney Disease Center The First Affiliated Hospital Zhejiang University School of Medicine Hangzhou 310003 China; ^2^ Key Laboratory of Kidney Disease Prevention and Control Technology Hangzhou Zhejiang 310003 China; ^3^ National Key Clinical Department of Kidney Diseases Hangzhou 310003 China; ^4^ Institute of Nephropathy Zhejiang University Hangzhou 310003 China; ^5^ Zhejiang Clinical Research Center of Kidney and Urinary System Disease Hangzhou 310003 China; ^6^ Cancer Institute of lntegrated Traditional Chinese and Western Medicine Key Laboratory of Cancer Prevention and Therapy Combining Traditional Chinese and Western Medicine, Zhejiang Key Laboratory of Disease‐Syndrome Integrated Cancer Prevention and Treatment Zhejiang Academy of Traditional Chinese Medicine Hangzhou Zhejiang 310012 China; ^7^ Department of General Surgery Sir Run‐Run Shaw Hospital Zhejiang University School of Medicine Hangzhou Zhejiang 310016 China; ^8^ Provincial Key Laboratory of Precise Diagnosis and Treatment of Abdominal Infection Sir Run‐Run Shaw Hospital Zhejiang University School of Medicine Hangzhou Zhejiang 310016 China

**Keywords:** chronic allograft rejection, macrophage‐myofibroblast transition, METTL3, renal fibrosis, TGF‐β1/Smad3

## Abstract

METTL3, a key enzyme in N6‐methyladenosine (m6A) modification, plays a crucial role in the progression of renal fibrosis, particularly in chronic active renal allograft rejection (CAR). This study explored the mechanisms by which METTL3 promotes renal allograft fibrosis, focusing on its role in the macrophage‐to‐myofibroblast transition (MMT). Using a comprehensive experimental approach, including TGF‐β1‐induced MMT cell models, METTL3 conditional knockout (METTL3 KO) mice, and renal biopsy samples from patients with CAR, the study investigates the involvement of METTL3/Smad3 axis in driving MMT and renal fibrosis during the episodes of CAR. We found that elevated m6A modification and METTL3 levels strongly correlated with enhanced MMT and increased fibrotic severity. METTL3 knockout (METTL3 KO) significantly increased the m6A modification of Smad3, decreased Smad3 expression, and inhibited M2‐driven MMT. Smad3 knockdown with siRNA (siSmad3) further inhibited M2‐driven MMT, while Smad3 overexpression rescued the inhibitory effects of METTL3 silencing, restoring M2‐driven MMT and fibrotic tissue damage. Additionally, the METTL3 inhibitor STM2457 effectively reversed M2‐driven MMT and alleviated fibrotic tissue damage in CAR. These findings highlight that METTL3 enhances M2‐driven MMT in renal fibrosis during CAR by promoting the TGF‐β1/Smad3 axis, suggesting that METTL3 is a promising therapeutic target for mitigating renal fibrosis in CAR.

## Graphical abstract

Schematic illustration of METTL3/Smad3 axis in M2‐driven MMT in CAR‐related fibrosis. By using several experimental models (including mouse BMDMs, mouse CAR models, and human renal biopsy samples), this study demonstrates that METTL3 enhances Smad3 levels to facilitate M2‐driven MMT and renal fibrosis in CAR.

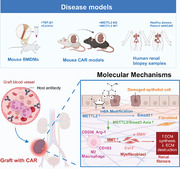

## 1 Introduction

Renal fibrosis represents the common pathological endpoint of chronic kidney diseases, characterized by the excessive accumulation of extracellular matrix (ECM) components that compromise renal structure and function.^[^ ^1^, ^2^, ^3^, ^4^ ^]^ Kidney transplantation is the preferred intervention for end‐stage kidney disease, however, post‐transplantation immune responses frequently lead to allograft dysfunction and failure.^[^ ^5^, ^6^, ^7^, ^8^, ^9^, ^10^ ^]^ Despite advancements in immunosuppressive therapies, long‐term renal allograft survival remains suboptimal, largely due to the occurrence of chronic active allograft rejection (CAR).^[^ ^7^, ^11^, ^12^, ^13^ ^]^ CAR often manifests as pathological damage distinguished by progressive renal fibrosis, marked by fibroblast proliferation, collagen deposition, epithelial‐mesenchymal transition (EMT), and macrophage‐to‐myofibroblast transition (MMT).^[^ ^14^, ^15^, ^16^ ^]^ Among these pathological changes, MMT has emerged as a critical contributor, particularly involving pro‐fibrotic M2 macrophages.^[^ ^17^, ^18^ ^]^ Emerging evidence reveals that M2 macrophages undergo MMT, transforming into myofibroblast‐like cells that actively secret ECM components and amplify the fibrotic response.^[^ ^19^, ^20^, ^21^, ^22^, ^23^ ^]^ The progression of renal fibrosis is driven by a complex interplay of signaling pathways, cytokines, growth factors, and alterations in gene expression and protein synthesis.^[^ ^24^, ^25^, ^26^, ^27^ ^]^ Among these, the transforming growth factor‐β (TGF‐β1)/Smad3 signaling pathway is central to initiating and sustaining fibrotic responses.^[^ ^28^, ^29^, ^30^, ^31^ ^]^ TGF‐β1, a potent fibrogenic cytokine, activates Smad3, which translocates to the nucleus to regulate genes involved in ECM synthesis and fibrosis.^[^ ^32^, ^33^ ^]^ Studies have demonstrated that inhibiting the TGF‐β1/Smad3 pathway can significantly reduce fibrosis and improve renal function, underscoring its importance as a therapeutic target in chronic kidney diseases.^[^ ^34^, ^35^, ^36^, ^37^ ^]^

In recent years, the role of N6‐methyladenosine (m6A) modification, a critical post‐transcriptional regulatory mechanism, has garnered attention for its influence on RNA metabolism and gene expression.^[^ ^38^, ^39^, ^40^ ^]^ The dynamic regulation of m6A modifications by m6A methyltransferases (“writers”), demethylases (“erasers”), and binding proteins (“readers”), has been implicated in diverse biological processes, including stem cell differentiation, DNA damage repair, inflammation, tumorigenesis, and immune response.^[^ ^41^, ^42^, ^43^, ^44^ ^]^ METTL3, the core m6A methyltransferase, catalyzes most methylations, with accumulating evidence linking its dysregulation to renal fibrosis.^[^ ^45^, ^46^, ^47^ ^]^ METTL3‐mediated m6A modification have been shown to upregulate fibrotic pathways, including SPRY1/ERK/NF‐kB and TGF‐β1/Smad3 signaling, correlating with disease severity.^[^ ^48^, ^49^, ^50^, ^51^ ^]^

Building on previous research in our center, which identified the Smad3 as a critical driver of M2 macrophage‐driven MMT (M2‐driven MMT) and renal fibrosis in CAR, this study further investigates the association of METTL3 with TGF‐β1/Smad3 signaling and its role in M2 driven MMT during CAR.^[^ ^52^ ^]^ By employing MMT cell models derived from mouse bone marrow‐derived macrophages (BMDMs), METTL3 conditional knockout (METTL3 KO) mice, and renal biopsy samples from patients with CAR, we demonstrate that METTL3 modulates the TGF‐β1/Smad3 pathway, facilitating M2 driven MMT and promoting fibrosis during CAR. Targeting METTL3, via siRNA, genetic knockout, or the small‐molecule inhibitor STM2457 alleviated M2‐driven MMT and tissue damage, highlighting METTL3 as a promising therapeutic target in CAR. Our findings establish a mechanistic link between METTL3 and renal allograft fibrosis, setting the stage for a shift in CAR management by integrating epigenetic regulation as a therapeutic approach to renal fibrosis.

## 2 Result

### 2.1 METTL3‐Mediated m6A Modification Involves MMT in Renal Allograft Rejection

**Figure 1:**
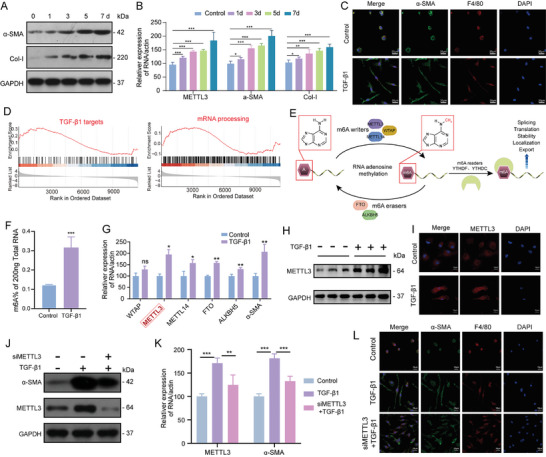
METTL3 Facilitates MMT Through m6A Modification in CAR. A,B) Time‐course expression of fibrotic markers α‐SMA and Col‐I at the protein and mRNA levels in 10 ng mL^−1^ TGF‐β1‐treated BMDMs over 1, 3, 5, and 7 days. C) Representative IF staining showing co‐expression of α‐SMA and F4/80 in control and TGF‐β1‐treated BMDMs. Scale bar = 20 µm. D) Gene set enrichment analysis of the prominent pathways in control and TGF‐β1‐treated BMDMs. E) Dynamic regulation of m6A modification in controlling RNA metabolism and function. F) Measurement of global m6A levels in control and TGF‐β1‐treated BMDMs. G) mRNA expression levels of key m6A regulators in control and TGF‐β1‐treated BMDMs. H) Protein levels of METTL3 in control and TGF‐β1‐treated BMDMs. I) IF analysis demonstrating METTL3 expression in control and TGF‐β1‐treated BMDMs. Scale bar = 10 µm. J,K) Protein and mRNA levels of METTL3 and α‐SMA in control, TGF‐β1‐treated, and siMETTL3 + TGF‐β1‐treated BMDMs. L) IF staining showing the co‐localization of METTL3 and α‐SMA in control, TGF‐β1‐treated, and siMETTL3+TGF‐β1‐treated BMDMs. Scale bar = 20 µm. ^*^ *p* < 0.05, ^**^ *p* < 0.01, ^***^ *p* < 0.001.

We first constructed MMT cell models using primary mouse BMDMs treated with TGF‐β1 for different periods. Time‐dependent increases in mRNA and protein levels of the fibrotic markers α‐SMA and Col‐I, confirm the successful induction of MMT (**Figure** **1**A,B). IF staining also verified MMT induction which showed co‐expression of macrophage marker F4/80 and α‐SMA and altered cell morphology (Figure 1C). We further applied RNA‐sequencing analysis to investigate the molecular changes accompanied by MMT. Gene set enrichment analysis (GSEA) identified significant alterations in several key pathways between control and MMT cells, encompassing TGF‐β1 pathways and processes related to mRNA processing (Figure 1D). Given the close relationship between m6A methylation modifications and RNA processing, we investigated changes in m6A levels in TGF‐β1‐induced MMT cells (Figure 1E). Consistent with the transcriptional changes, MMT cells have significantly higher global m6A modification levels compared to control cells, accompanied by an upregulation of m6A‐associated regulatory factors (Figure 1F). Notably, the expression of the key m6A methyltransferase METTL3 was markedly elevated in TGF‐β1‐ induced MMT cells (Figure 1G). WB and IF staining further confirmed the high expression of METTL3 in TGF‐β1‐induced MMT cells (Figure 1H–I). To explore the role of METTL3 in MMT during renal fibrosis, small interfering RNA transfection (siRNA)was used to knock down METTL3 expression in MMT cells. WB results showed that α‐SMA level was lower in METTL3 siRNA (siMETTL3) MMT cells than in control cells and MMT cells (Figure 1J). RT‐qPCR also revealed the suppressed α‐SMA expression in siMETTL3 MMT cells (Figure 1K). Additionally, IF staining showed that siMETTL3 inhibited the TGF‐β1‐induced increase in α‐SMA expression (Figure 1L). These findings highlight the crucial role of METTL3‐mediated m6A modification in promoting MMT and contributing to renal fibrosis during CAR.

### 2.2 High m6A Modification Level is Associated with Renal Fibrosis During CAR in Vivo

**Figure 2:**
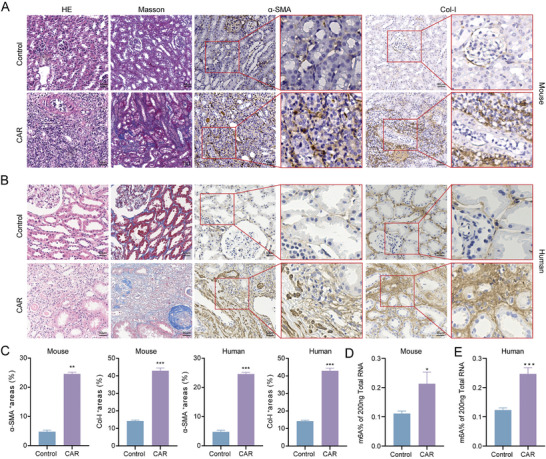
CAR Exhibits Serious Renal Fibrosis and a High Level of m6A Modification. A) Representative histological images (HE and Masson staining) and IHC staining for fibrotic markers α‐SMA and Col‐I in kidney tissues from control and CAR mice. Scale bar = 50 µm. B) Representative histological images (HE and Masson) and IHC staining for fibrotic markers α‐SMA and Col‐I in human renal biopsy samples from healthy controls and patients with CAR. Scale bar = 50 µm. C) Quantitative IHC analysis of α‐SMA and Col‐I in both mouse and human renal biopsy samples. D,E) m6A modification levels in kidney tissues from control and CAR mice as well as healthy controls and patients with CAR. ^*^ *p* < 0.05, ^**^ *p* < 0.01, ^***^ *p* < 0.001.

Besides the involvement of m6A Modification in the MMT cells, we further investigated the in vivo effects of m6A modifications on renal fibrosis during CAR using both mouse and human renal biopsy samples. Histopathological analysis revealed that CAR mice exhibited significant fibrotic kidney tissue damage compared to control mice, including tubular atrophy, thickening of the tubular basement membrane, interstitial fibrosis with inflammatory cell infiltration, and severe collagen deposition in the renal interstitium. IHC staining also exhibited intense α‐SMA and Col‐I on kidney tissues of CAR mice (**Figure** **2**A). To validate these findings, we performed parallel staining analyses on human renal biopsy samples from patients with CAR. Consistently, human specimens exhibited severe fibrotic tissue damage, characterized by significant interstitial fibrosis, extensive collagen deposition, and strong α‐SMA and Col‐I staining (Figure 2B). Quantitative IHC analysis further confirmed significantly increased expression of α‐SMA and Col‐I in both CAR mice and patients with CAR, underscoring the occurrence of renal fibrosis during CAR and the translational relevance of the CAR mouse model to human CAR (Figure 2C). Additionally, we detected the level of m6A RNA modification in total RNA extracted from kidney tissues of CAR mice and patients with CAR. Both groups exhibited significantly higher m6A levels compared to their respective controls (Figure 2D,E). This tendency aligned with the results in vitro, emphasizing the significance of m6A modification in driving MMT and renal fibrosis during CAR.

### 2.3 Upregulated METTL3 Expression Enhances MMT and Renal Fibrosis in Both Mouse CAR Models and Human Renal Biopsy Samples From CAR Patients

**Figure 3:**
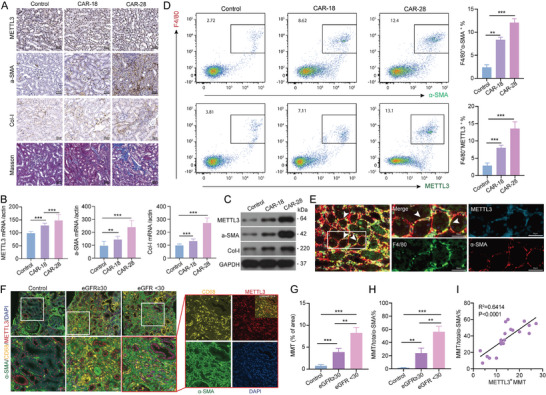
METTL3 Expression Correlates with MMT and the Degree of Fibrosis in CAR. A) Masson and IHC staining for METTL3, α‐SMA, and Col‐I in kidney tissues from control, CAR‐18, and CAR‐28 mice. Scale bar = 50 µm. B,C) mRNA and protein levels of METTL3, α‐SMA, and Col‐I in kidney tissues from control, CAR‐18, and CAR‐28 mice. D) Flow cytometry analysis identifying F4/80^+^ α‐SMA^+^ and F4/80^+^METTL3^+^ cells in kidney tissues from control, CAR‐18, and CAR‐28 mice. Quantitative analysis of F4/80^+^ α‐SMA^+^ and F4/80^+^ METTL3^+^ populations in kidney tissues from control, CAR‐18, and CAR‐28 mice. E) Representative IF staining showing the colocalization of METTL3, α‐SMA, and F4/80 in kidney tissues from CAR‐28 mice. Scale bar = 50 µm. F) Representative IF staining of METTL3, α‐SMA, and CD68 in renal biopsy samples from healthy controls, patients with eGFR ≥ 30, and patients with eGFR < 30. Scale bar = 50 µm. G,H) Quantitative analysis of MMT cells (co‐expressing α‐SMA and CD68) and their percentage in total α‐SMA^+^ populations in renal biopsy samples from healthy controls, patients with eGFR ≥ 30, and patients with eGFR < 30. I) Correlation of METTL3^+^MMT with the proportion of MMT in total α‐SMA^+^ populations. ^*^ *p* < 0.05, ^**^ *p* < 0.01, ^***^ *p* < 0.001.

Given that METTL3 serves as a key component of m6A modification and its remarkable increase in the MMT cells, we next focused on METTL3 to explore the mechanism behind the increased m6A modification with renal fibrosis. IHC analysis exhibited that METTL3 was expressed at low levels in the kidney tissues of sham mice (*n* = 6) (named control group) but was strongly upregulated in kidney tissues of CAR mice on post‐transplantation days 18 (*n* = 6) and 28 (*n* = 6) (named CAR‐18 and CAR‐28 groups, respectively). This increase in METTL3 expression was accompanied by a concomitant increase in collagen deposition and α‐SMA and Col‐I levels (**Figure** **3**A). RT‐qPCR and WB analysis also revealed the progressive increases in METTL3, α‐SMA, and Col‐I among control, CAR‐18, and CAR‐28 groups at both mRNA and protein levels (Figure 3B,C). Flow cytometry analysis of kidney tissues further demonstrated the increase in the number of cells co‐expressing F4/80 and α‐SMA as well as F4/80 and METTL3 among control, CAR‐18, and CAR‐28 groups (Figure 3D). IF staining of METTL3, F4/80, and α‐SMA colocalization indicated that METTL3 was predominantly expressed in MMT cells within the kidney tissues of CAR‐28 mice (Figure 3E). These results suggest that the upregulation of METTL3 contributed to the MMT‐driven renal fibrosis during CAR.

To validate the results obtained from the mouse CAR model, we applied clinical biopsy specimens from patients with CAR to assess the presence of MMT cells (co‐expressing CD68 and α‐SMA) and the METTL3 expression across different extents of renal fibrosis. IF staining revealed extensive fibrotic damage in most patients with CAR, with a marked increase in the percentage of METTL3^+^ MMT cells as the disease progressed (Figure 3F). Notably, renal biopsies from patients with an estimated glomerular filtration rate (eGFR) <30 showed significant infiltration of METTL3^+^ MMT cells, indicating a strong association between advanced renal dysfunction and MMT. As the disease progressed with decreasing eGFR levels, the severity of fibrotic tissue damage increased along with a rise in the number of MMT cells among fibrotic tissues and the proportion of MMT cells among α‐SMA^+^ myofibroblasts (Figure 3G,H). Moreover, the percentage of METTL3^+^ MMT cells was positively correlated with the proportion of MMT cells among α‐SMA^+^ myofibroblasts (Figure 3I). These results provide strong evidence that METTL3 contributes to MMT‐driven renal fibrosis in CAR, particularly in the advanced stages of the disease.

### 2.4 METTL3 Knockout Attenuates MMT and Renal Fibrosis During CAR

**Figure 4:**
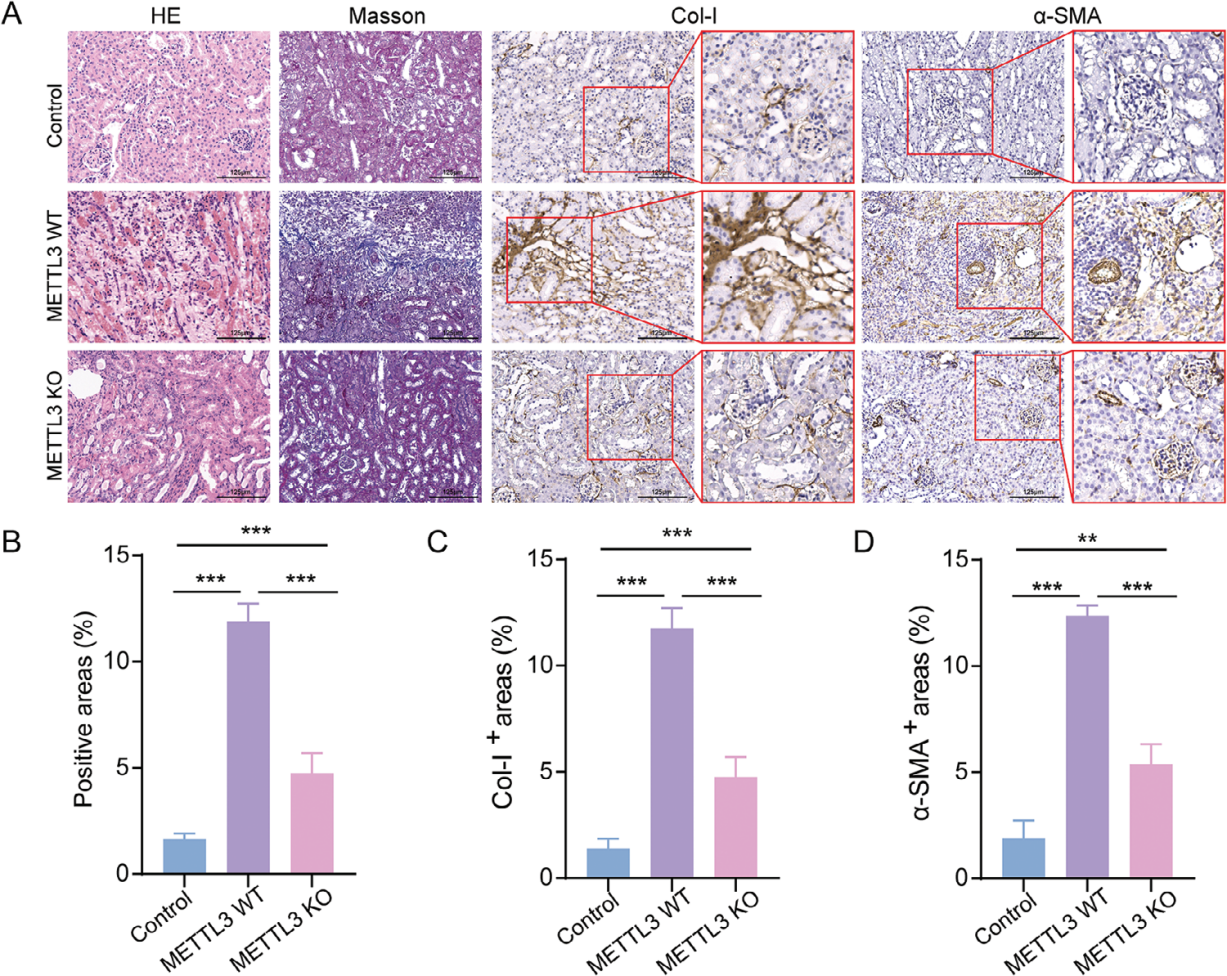
METTL3 Knockout Inhibits MMT and Improves Fibrotic Tissue Damage During CAR. A) HE, Masson, and IHC staining for α‐SMA and Col‐I of kidney tissues from control, METTL3 WT, and METTL3 KO mice. Scale bar = 125 µm. B–D) Quantitative analysis of collagen deposition area in Masson and the expression of α‐SMA and Col‐I in IHC staining for kidney tissues from control, METTL3 WT, and METTL3 KO mice. ^*^ *p* < 0.05, ^**^ *p* < 0.01, ^***^ *p* < 0.001.

**Figure 5:**
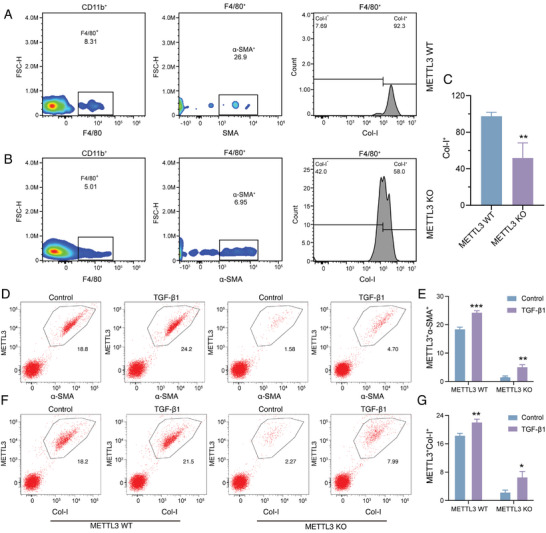
METTL3 Knockout Reduces the number of MMT cells in CAR. A–C) Identification and quantification of MMT cells in kidney tissues from METTL3 WT and METTL3 KO mice. D,E) Identification and quantification of METTL3^+^ α‐SMA^+^ MMT cells in TGF‐β1‐treated BMDMs from METTL3 KO and METTL3 WT mice. F,G) Identification and quantification of METTL3^+^ Col‐I^+^ MMT cells in TGF‐β1‐treated BMDMs from METTL3 WT and METTL3 KO mice. ^*^ *p* < 0.05, ^**^ *p* < 0.01, ^***^ *p* < 0.001.

To elucidate the direct role of METTL3 in renal fibrosis during CAR, we utilized sham‐operated (named control group) and kidney transplantation mouse models with METTL3 wild‐type (named METTL3 WT group) and knockout (named METTL3 KO group). Histological staining of the transplanted kidney tissues in METTL3 WT showed a significantly larger area of fibrosis than the control group, which confirmed the occurrence of CAR. Compared to METTL3 WT and control mice, METTL3 KO mice exhibited significantly reduced fibrotic tissue damage, collagen deposition, and expression of Col‐I and α‐SMA (**Figure** **4**A). Quantitative analyses validated marked decreases in collagen deposition, Col‐I, and α‐SMA levels in METTL3 KO mice (Figure 4B–D). We further utilized flow cytometry to analyze the percentage of MMT cells co‐expressing F4/80 with either α‐SMA or Col‐I in kidney tissues from METTL3 WT and METTL3 KO mice. The results indicated that METTL3 KO mice had significantly fewer MMT cells compared to METTL3 WT mice (**Figure** **5**A–C). Moreover, we isolated BMDMs from METTL3 WT and KO mice for in vitro experiments. Consistent with results of tissue experimental results, METTL3 KO markedly inhibited the proportion of MMT cells (Figure 5D–G). Cumulatively, these findings highlight METTL3 as a direct driver for MMT in CAR.

**Figure 6:**
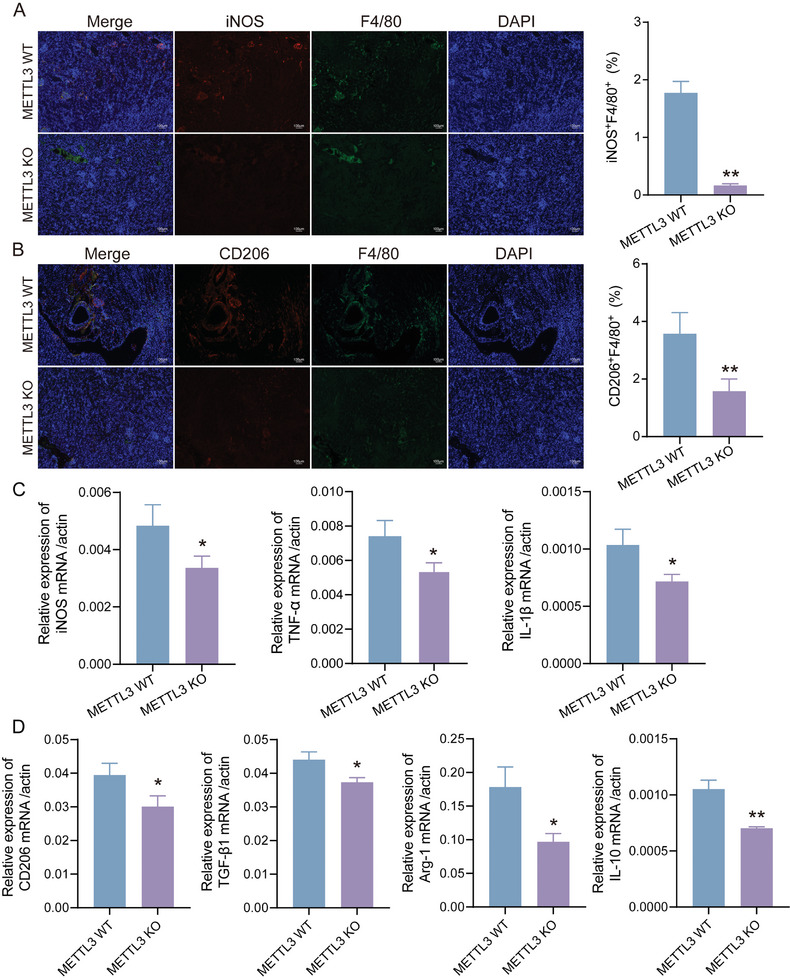
METTL3 Knockout Inhibits Macrophage Infiltration in Renal Allografts During CAR. A,B) IF staining and quantitative analysis of kidney tissues from METTL3 WT and METTL3 KO mice showing M1 macrophage marker (iNOS) and M2 macrophage marker (CD206). Scale bar = 100 µm. C,D) mRNA levels of M1 macrophage markers (iNOS, TNF‐α, and IL‐1β) and M2 macrophage markers (CD206, TGF‐β1, Arg‐1, and IL‐10) in kidney tissues from METTL3 WT and METTL3 KO mice. ^*^ *p* < 0.05, ^**^ *p* < 0.01, ^***^ *p* < 0.001.

Additionally, IF analysis with markers for M1 (iNOS) and M2 (CD206) macrophages revealed that METTL3 KO mice had reduced infiltration of both M1 and M2 macrophage subsets in kidney tissues compared to METTL3 WT mice (**Figure** **6**A,B). RT‐qPCR analysis of M1 (iNOS, TNF‐α, IL‐1β) and M2 (CD206, TGF‐β1, Arg‐1, IL‐10) macrophage markers also supported these findings, confirming that METTL3 KO attenuated the infiltration of both macrophage subsets (Figure 6C,D).

### 2.5 METTL3 Mainly Strengthens M2‐Driven MMT During CAR in Both Mouse CAR Models and Human Renal Biopsy Samples From CAR Patients

**Figure 7:**
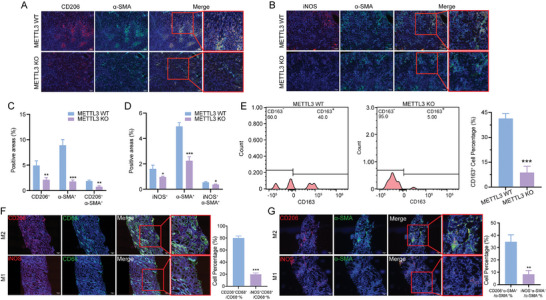
METTL3 Knockout Decreases MMT Predominantly Originating from M2 Macrophages in CAR. A,B) Representative IF staining of CD206 and α‐SMA in kidney tissues from METTL3 WT and METTL3 KO mice. Scale bar = 100 µm. C,D) Quantitative analysis of the CD206^+^, α‐SMA^+^, and CD206^+^ α‐SMA^+^ cell populations and the iNOS^+^, α‐SMA^+^, and iNOS^+^ α‐SMA^+^ cell populations in kidney tissues from METTL3 WT and METTL3 KO mice. E,F) Identification and quantitation of CD163^+^ F4/80^+^ α‐SMA^+^ MMT cells in kidney tissues from METTL3 WT and METTL3 KO mice. F) Representative IF staining of iNOS, CD206, and CD68 in renal biopsy samples from patients with CAR. Scale bar = 50 µm. Quantitative analysis of CD206^+^ CD68^+^ and iNOS^+^ CD68^+^ cells as a percentage of the total CD68^+^ macrophage population in these human renal biopsy samples. G) Representative IF staining of iNOS, CD206, and α‐SMA in renal biopsy samples from patients with CAR. Scale bar = 100 µm. Quantitative analysis of the percentage of CD206^+^ α‐SMA^+^ and iNOS^+^ α‐SMA^+^ cells accounting for the total MMT cell population in these human renal biopsy samples. ^*^ *p* < 0.05, ^**^ *p* < 0.01, ^***^ *p* < 0.001.

To further explore the specific macrophage subsets contributing to MMT under METTL3 regulation, we examined M1 (iNOS) and M2 macrophage (CD206) markers in MMT cells from kidney tissues of METTL3 WT and KO mice. IF staining revealed that MMT cells predominantly expressed the M2 macrophage marker CD206, with minimal M1 marker iNOS expression, suggesting that MMT cells mainly originated from M2 macrophages during CAR (**Figure** **7**A,B). Quantitative analysis showed that METTL3 KO mice exhibited a significantly reduced number of CD206^+^ MMT and iNOS^+^ MMT cells compared to METTL3 WT mice (Figure 7C,D). Flow cytometry results further confirmed that the proportion of CD163^+^ MMT cells, another M2 macrophage marker, was lower in the kidney tissues of METTL3 KO mice compared to METTL3 WT mice (Figure 7E).

We further validated the findings observed in the mouse CAR models using human renal biopsy samples from patients with CAR. IF staining revealed a significant presence of M2 macrophages (cells co‐expressing CD206 and CD68) with markedly fewer M1 macrophages (cells co‐expressing iNOS and CD68) (Figure 7F).^[^ ^52^ ^]^ Quantitative analysis confirmed that CD206^+^ cells made up the majority of the CD68^+^ macrophage population (Figure 7F). Furthermore, IF staining also showed that most MMT cells exhibited high expression of the M2 marker CD206 and low expression of the M1 marker iNOS (Figure 7G). Quantitative analysis revealed that CD206^+^ MMT cells accounted for a larger proportion of the total MMT cell population compared to those expressing iNOS (Figure 7G). The consistent results observed in both the mouse models and human samples demonstrated that METTL3 contributed to M2‐driven MMT during CAR.

### 2.6 Smad3 Serves as a Key Downstream Effector of METTL3‐Induced MMT and Fibrosis During CAR

**Figure 8:**
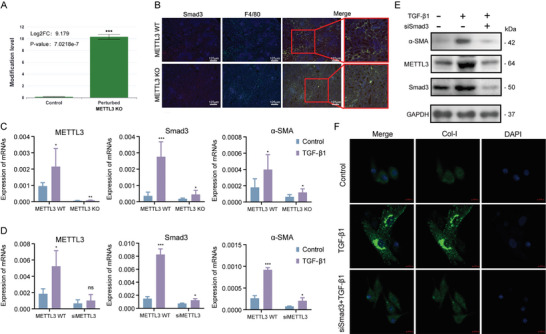
Smad3 as the Downstream of METTL3 For driving MMT and Renal Fibrosis in CAR. A) m6A modification levels of Smad3 between control and METTL3 KO groups. B) IF staining of Smad3 and F4/80 coexpression in kidney tissues from METTL3 WT and METTL3 KO mice. Scale bar = 125 µm. C) mRNA levels of METTL3, Smad3, and α‐SMA in TGF‐β1‐treated BMDMs from METTL3 WT and METTL3 KO mice. D) mRNA levels of METTL3, Smad3, and α‐SMA in TGF‐β1‐treated BMDMs from METTL3 WT and METTL3 Knockdown (siMETTL3) mice. E) Protein levels of METTL3, Smad3, and α‐SMA in control, TGF‐β1‐treated, and TGF‐β1+siSmad3 BMDMs from METTL3 WT mice. F) IF staining of Col‐I expression in control, TGF‐β1‐treated, and TGF‐β1+siSmad3 BMDMs from METTL3 WT mice. Scale bar = 10 µm. ^*^ *p* < 0.05, ^**^ *p* < 0.01, ^***^ *p* < 0.001.

The TGF‐β1/Smad3 pathway is pivotal in orchestrating macrophage infiltration, M2 polarization, and MMT during fibrogenesis.^[^ ^53^, ^54^, ^55^, ^56^, ^57^ ^]^ To investigate whether METTL3 involved in TGF‐β1/Smad3‐mediated fibrogenesis, we first used the M6A2Target database (http://m6a2target.canceromics.org) to predict the influence of METTL3 on Smad3.^[^ ^58^ ^]^ The results suggested that METTL3 could influence Smad3, which was confirmed by increased m6A modification of Smad3 after METTL3 KO in mouse BMDMs (RM2Target ID: 1490074) (**Figure** **8**A). To determine whether Smad3 is modified by METTL3 during MMT, we examined Smad3 expression in macrophages within kidney tissues of METTL3 WT and METTL3 KO mice. IF analysis showed strong Smad3 expression in macrophages from METTL3 WT mice compared to METTL3 KO mice (Figure 8B). To further explore the modulation of Smad3 by METTL3 during MMT, BMDMs from METTL3 WT and KO mice were stimulated with TGF‐β1 to induce MMT cells in vitro. MMT cells from METTL3 KO mice exhibited reduced mRNA levels of α‐SMA, METTL3, and Smad3 compared to those from METTL3 WT mice (Figure 8C). Knockdown of METTL3 with siMETTL3 in TGF‐β1 induced‐BMDMs from METTL3 WT mice similarly reduced Smad3, α‐SMA, and Col‐I expression during MMT (Figure 8D). To confirm the role of Smad3 in METTL3‐mediated MMT, we isolated BMDMs from METTL3 WT mice and divided them into three experimental conditions: control (untreated BMDMs), TGF‐β1 (BMDMs treated with TGF‐β1 to induce MMT), and TGF‐β1+ siSmad3 (BMDMs transfected with Smad3‐specific siRNA, followed by TGF‐β1 treatment). WB results demonstrated that TGF‐β1 treatment increased the expression of α‐SMA, METTL3, and Smad3. Notably, Smad3 knockdown via siSmad3 effectively reduced α‐SMA levels in METTL3 WT BMDMs, confirming the role of Smad3 as a key downstream effector in METTL3‐mediated MMT (Figure 8E). Consistent with the WB findings, IF revealed increased Col‐I staining following TGF‐β1 treatment, which was attenuated by siSmad3 (Figure 8F). These findings suggested that Smad3 is directly involved in MMT mediated by METTL3. Smad3 knockdown protects METTL3 WT BMDMs against TGF‐β1‐induced MMT, thereby strengthening the hypothesis that METTL3 promotes MMT via Smad3.

### 2.7 METTL3 Elevates Smad3 Level to Enhance M2‐Driven MMT and Renal Fibrosis in CAR

**Figure 9:**
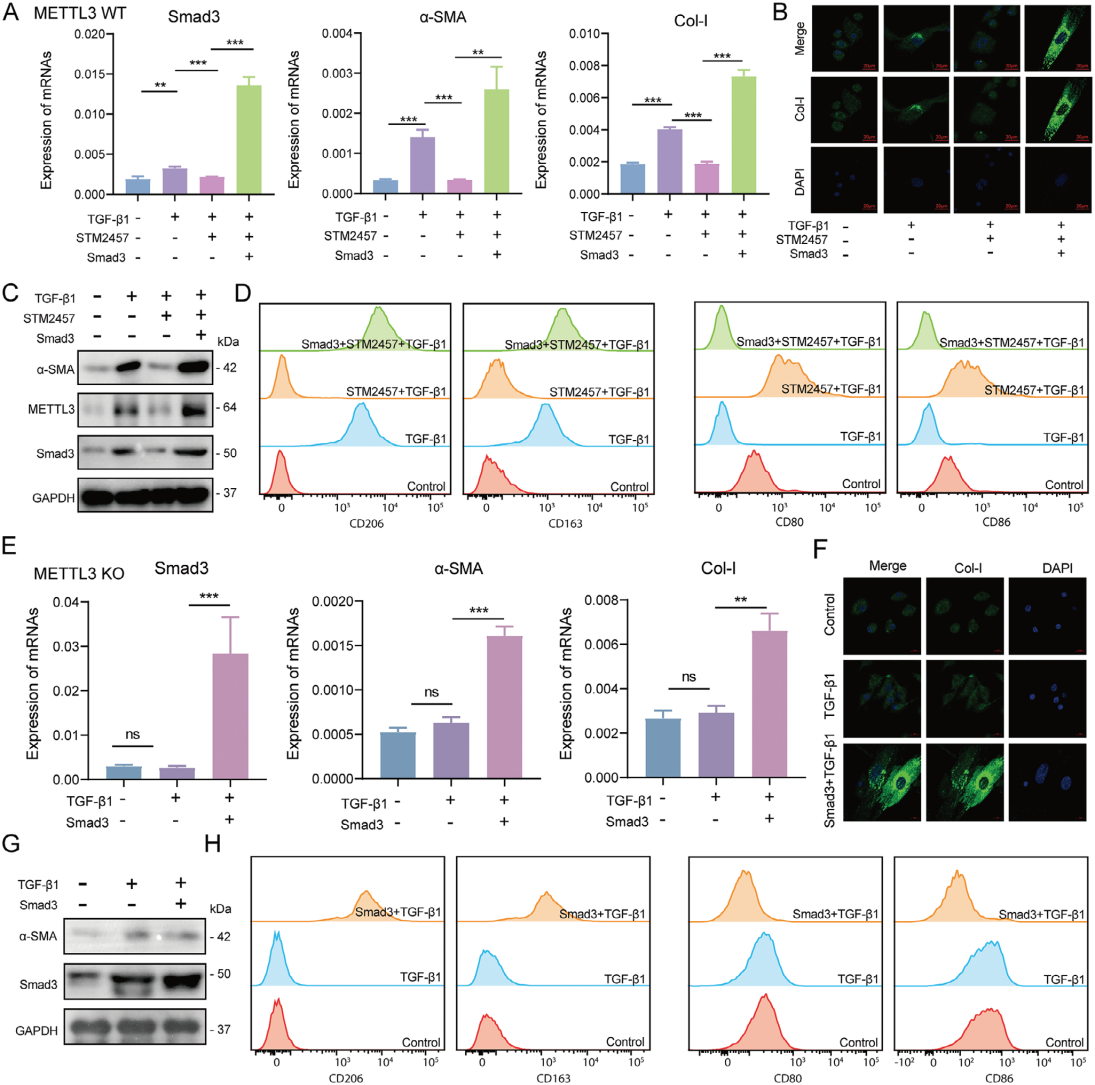
METTL3/Smad3 axis Promotes M2‐driven MMT in Renal Fibrosis During CAR. A) mRNA levels of Smad3, α‐SMA, and Col‐I in BMDMs from METTL3 WT mice with or without TGF‐β1, STM2457 treatment, or Smad3 overexpression. B) IF staining of Col‐I expression in BMDMs from METTL3 WT mice among control, TGF‐β1‐treated, TGF‐β1+ STM2457, TGF‐β1+ STM2457+ Smad3 overexpression groups. Scale bar = 20 µm. C) Protein levels of α‐SMA, METTL3, Smad3, and Col‐I in BMDMs from METTL3 WT among control, TGF‐β1‐treated, TGF‐β1+ STM2457, TGF‐β1+ STM2457+ Smad3 overexpression groups. D) Number changes of M2 (CD206 and CD163) and M1 (CD80 and CD86) macrophages from METTL3 WT mice among control, TGF‐β1‐treated, TGF‐β1+ STM2457, TGF‐β1+ STM2457+ Smad3 overexpression groups. E) mRNA levels of Smad3, α‐SMA, and Col‐I in BMDMs from METTL3 KO mice among control, TGF‐β1‐treated, TGF‐β1+ Smad3 overexpression groups. F) IF staining of Col‐I expression in BMDMs from METTL3 KO mice among control, TGF‐β1‐treated, TGF‐β1+ Smad3 overexpression groups. Scale bar = 10 µm. G) Protein levels of Smad3 and α‐SMA in BMDMs from METTL3 KO mice among control, TGF‐β1‐treated, TGF‐β1+ Smad3 overexpression groups. H) Number changes of M2 (CD206 and CD163) and M1 (CD80 and CD86) macrophages from METTL3 KO mice among control, TGF‐β1‐treated, TGF‐β1+ Smad3 overexpression groups. ^*^ *p* < 0.05, ^**^ *p* < 0.01, ^***^ *p* < 0.001.

To further validate the METTL3/Smad3 axis, we conducted rescue experiments in METTL3 WT and KO BMDMs. Treatment of METTL3 WT BMDMs with the METTL3 specific inhibitor STM2457 during TGF‐β1‐stimulated MMT in BMDMs from METTL3 WT mice inhibited Smad3, α‐SMA, and Col‐I mRNA expression (**Figure** **9**A). WB and IF analyses consistently showed that STM2457 disrupted the TGF‐β1‐induced MMT, while Smad3 overexpression rescued the effects of METTL3 inhibition (Figure 9B,C). We further explored the role of the METTL3/Smad3 axis in M2‐driven MMT using flow cytometry to assess M2 (CD206, CD163) and M1 (CD80, CD86) marker expression. METTL3 inhibitor STM2457 reduced M2 macrophage marker expression (CD206, CD163) and disrupted the MMT. While Smad3 overexpression with STM2457 rescued the M2 marker expression and restored the MMT (Figure 9D). Interestingly, TGF‐β1 stimulation alone in METTL3 KO BMDMs did not affect Smad3, α‐SMA, or Col‐I expression at both mRNA and protein levels, but Smad3 overexpression rescued the reduced levels of α‐SMA and Col‐I (Figure 9E–G). Furthermore, METTL3 KO disrupted TGF‐β1‐induced MMT, while Smad3 overexpression in METTL3 KO BMDMs restored M2 marker expression and rescued the MMT (Figure 9H). The changes in M2 macrophage marker expression align with the alterations in the number of MMT cells, further confirming that M2 macrophages are the primary subsets driving MMT. These findings underscored the critical role of the METTL3/Smad3 axis in mediating M2‐driven MMT during CAR‐associated fibrosis.

## 3 Discussion

Our research underscores the critical role of METTL3 in modulating TNF‐β/Smad3 signaling and its downstream effects on MMT and the development of renal fibrosis in CAR. M2 macrophages, known for their pro‐fibrotic properties, undergo MMT and transform into myofibroblast‐like cells that secrete ECM components to exacerbate fibrosis. The strong correlation among METTL3, M2‐driven MMT, and fibrotic tissue damage of allografts further emphasizes the importance of METTL3 and MMT in the fibrotic process, making it a promising therapeutic target to mitigate fibrosis and fibrosis‐related renal dysfunction during CAR.

m6A is the most abundant RNA modification and plays a critical role in various cellular processes and the progression of multiple diseases. Increasing evidence has highlighted the close connection between m6A methylation and renal fibrosis in various kidney diseases.^[^ ^42^, ^59^, ^60^ ^]^ As a core enzyme of m6A modification, METTL3 participates in numerous physiological and pathological processes, including the progression of fibrosis.^[^ ^45^, ^48^, ^61^ ^]^ Elevated m6A levels have been observed in models of acute kidney injury and ischemia‐reperfusion injury, with increased expression of m6A regulators such as METTL3, METTL14, FTO, and ALKBH5.^[^ ^62^, ^63^ ^]^ In renal fibrosis models, models of renal fibrosis, such as unilateral ureteral obstruction (UUO) and TGF‐β1‐induced EMT in HK2 cells, increased m6A levels and METTL3 activation have been shown to enhance fibrotic processes.^[^ ^51^ ^]^ Recent studies have also revealed that METTL3 expression is upregulated in CKD patients, with enriched m6A modification targeting the cGAS‐STING signaling pathway, thereby promoting disease progression. Furthermore, antisense oligonucleotides targeting METTL3 expression have demonstrated potential for alleviating renal fibrosis.^[^ ^64^ ^]^

Despite these findings, it remains unclear whether m6A modification influences the development of CAR in human kidney transplantation and whether targeting m6A can mitigate renal fibrosis progression during the occurrence of CAR. Our study provides evidence from cellular, animal, and human models, demonstrating that increased m6A modification and METTL3 expression are strongly associated with enhanced MMT and renal fibrosis during CAR.

Our previous research has established that MMT is a key mechanism underlying myofibroblast accumulation during CAR and that M2 macrophages are the primary macrophages involved in this process.^[^ ^52^ ^]^ Given the pro‐fibrotic properties, M2 macrophages undergo MMT, transforming into myofibroblast‐like cells and secreting ECM components to exacerbate fibrosis. However, the direct connection between m6A modification and MMT during CAR remains elucidated. In this study, we identified a strong correlation between METTL3, M2‐driven MMT, and fibrotic tissue damage in allografts, underscoring the importance of METTL3 and MMT in the progression of fibrosis. In both the CAR mouse model and renal biopsy samples from CAR patients, METTL3 was confirmed to drive M2‐dominated MMT, thereby exacerbating tissue fibrotic damage. Upregulation of METTL3 was found to promote the activation of Smad3, leading to the overexpression of fibrosis‐related molecules such as Col‐I and α‐SMA. Functional validation experiments demonstrated that silencing METTL3 using siRNA, genetic knockout, or treatment with the inhibitor STM2457 significantly reduced Smad3 expression, disrupted M2‐driven MMT, and alleviated renal fibrosis during CAR.

Our findings extend previous studies by revealing that METTL3 promotes M2 macrophage‐driven MMT and enhances fibrosis in allografts by regulating the TGF‐β1/Smad3 pathway. As a central driver of fibrosis, the TGF‐β1/Smad3 axis orchestrates the expression of fibrotic genes and ECM synthesis.^[^ ^65^, ^66^, ^67^ ^]^ Inhibition of this pathway has consistently demonstrated the potential to reduce fibrosis and improve kidney function in various preclinical models of kidney disease.^[^ ^68^, ^69^, ^70^ ^]^ While the TGF‐β1/Smad3 pathway plays a core role in fibrosis, fibrotic processes are complex and regulated by multiple interconnected signaling networks. Other inflammatory and fibrotic pathways, such as MAPK/NF‐κB signaling, Wnt/β‐catenin cascade, PI3K/Akt signaling, or the hypoxia‐inducible factor‐1α (HIF‐1α) pathway, may act in concert with TGF‐β1/Smad3 to regulate fibrosis, adding to the complexity of fibrotic progression.^[^ ^71^, ^72^, ^73^, ^74^ ^]^ Future studies exploring these complementary mechanisms will provide a more nuanced understanding of METTL3's role in renal fibrosis and its broader implications.

In addition, we have also confirmed that inhibition of METTL3 via siRNA, genetic knockout, or the small‐molecule inhibitor STM2457 effectively alleviated MMT and reduced fibrotic tissue damage during CAR. These findings highlight the critical role of METTL3 as a key regulator of MMT and a promising therapeutic target for addressing fibrosis in CAR. Future investigations into METTL3‐targeted interventions, including small‐molecule inhibitors and RNA‐based therapies, may pave the way for novel strategies to mitigate fibrotic tissue damage and enhance long‐term allograft survival. Beyond targeting METTL3 alone, combining its inhibition with TGF‐β inhibitors or anti‐inflammatory therapies could increase synergistic effects in reducing fibrosis and mitigating CAR. Moreover, integrating complementary inflammatory and fibrotic pathways, such as ferroptosis, NF‐κB, and cGAS‐STING signaling, into the METTL3/Smad3 axis offers a comprehensive perspective for developing multifaceted therapeutic approaches for CAR and its associated fibrosis.^[^ ^61^ ^]^

In conclusion, our study provides novel insights into the mechanisms underlying MMT and fibrosis progression during CAR from an epitranscriptomic perspective. We first reveal that METTL3 facilitates M2‐driven MMT by modulating the TGF‐β1/Smad3 pathway, contributing to renal fibrosis in CAR. These findings offer a promising therapeutic target for renal fibrosis and pave the way for the development of innovative treatments to improve allograft outcomes.

## 4 Experimental Section

### Human Renal Allograft Biopsy Specimens

Renal allograft biopsy specimens were collected from patients diagnosed with chronic active kidney transplant rejection (CAR) at the Kidney Disease Center of the First Affiliated Hospital, Zhejiang University School of Medicine, between September 2021 and September 2022. Patients with CAR included in the study (*n* = 20) met the following criteria: a confirmed clinical diagnosis of chronic active renal rejection, the pathological diagnosis of chronic active rejection according to the Banff 2011 criteria, and an estimated glomerular filtration rate (eGFR) <60 mL min^−1^/1.73 m^2^. Control samples (*n* = 20) were obtained from healthy donor kidney tissues collected at the time of transplantation, with no underlying renal pathology. All biopsy specimens were collected using standardized percutaneous biopsy techniques and preserved in formalin for histological analysis or flash‐frozen for molecular studies. This study was approved by the Ethics Committee of Zhejiang University (Ethical Approval No. 2022422) and informed consent was obtained from all participants prior to sample collection. The study adhered to institutional ethical guidelines and ensured the confidentiality of patient data throughout the research process.

### Animal Studies

BALB/c and C57BL/6 male mice aged 10–12 weeks and weighing 25–28 g were sourced from Gempharmatech Co., Ltd. (Nos. SCXK(SU)2023‐0009) and maintained on standard chow and conditions prior to the experiments. A CAR model was established by transplanting kidneys from BALB/c mice into C57BL/6 mice. Control kidney transplants were performed between C57BL/6 mice. Sham‐operated mice underwent the same surgical procedures without kidney transplantation (*n* = 6). To investigate the role of METTL3 in this model, METTL3^Flox/Flox^ mice (Gempharmatech Co., Ltd, Nanjing, China (Nos. SCXK(SU)2023‐0009)) were crossed with LYZ2^Cre^ mice (Gempharmatech Co., Ltd, Nanjing, China (Nos. SCXK(SU)2023‐0009)) to generate macrophage‐specific METTL3 knockout (METTL3 KO; METTL3^+/+^LYZ2^CRE+/−^) mice (*n* = 6). Wild‐type mice without METTL3 knockout are referred to as METTL3 WT (METTL3^+/+^LYZ2^CRE−/−^) (*n* = 6). Anesthesia was induced with 3–5% isoflurane and maintained at 1.5% throughout the procedure. Kidney grafts were transplanted after left kidney nephrectomy, with vascular reconnection to the abdominal aorta and inferior caval vein, and the ureter reconnected to the bladder dome. Total ischemic time was ≈35–40 min.^[^ ^75^, ^76^ ^]^ After surgery, mice received 1 mg kg^−1^ day^−1^ FK506 intraperitoneally for 28 days, while control mice received saline. Kidneys were harvested for analysis on Day 18 (*n* = 6) and 28 (*n* = 6), respectively. The Animal Experimental Ethics Committee of The First Affiliated Hospital, Zhejiang University School of Medicine (project number 2022646) approved all procedures, and the study followed the Guide for the Care and Use of Laboratory Animals to ensure ethical standards.

### Cell Lines and Culture Conditions

Primary BMDMs were isolated from mouse bone marrow and cultured in DMEM/F12 supplemented with 10% heat‐inactivated fetal bovine serum (FBS) and 50 ng mL^−1^ macrophage colony‐stimulating factor (M‐CSF, Gibco) for 7 days to induce macrophage differentiation. For MMT, BMDMs were treated with 10 ng mL^−1^ TGF‐β1 for up to 7 days, with the medium refreshed every 48 h. METTL3 expression was knocked down using 20 nm small interfering RNA (siRNA) from Genepharm (Shanghai, China), or METTL3 activity was inhibited with 10 µm STM2457 (Chemicals, DC53045, Shanghai, China). BMDMs isolated from METTL3 KO mice were also treated with TGF‐β1, and Smad3 overexpression was induced via plasmid transfection.

### Renal Morphology

To evaluate renal fibrotic morphology, paraffin‐embedded kidney samples were sectioned at 3 µm thickness and stained using hematoxylin eosin (HE), and Masson's trichrome staining to assess tissue damage and fibrotic regions. Photographs were taken from at least three randomly selected fields per kidney segment at 20× magnification. Areas of fibrosis were quantitatively assessed as the percentage of fibrotic area relative to the total tissue area using ImageJ 1.52d software, with thresholding applied to highlight fibrotic regions.

### Immunohistochemistry (IHC) and Immunofluorescence (IF) Staining

Tissue sections were treated with primary antibodies targeting F4/80, α‐SMA, METTL3, collagen I (Col‐I), CD206, iNOS, CD163, and CD68 (CST‐Cell Signaling Technology, Danvers, MA, USA), with antibody concentrations/dilutions according to the manufacturer's protocols.^[^ ^77^ ^]^ Immunofluorescence images were captured using a Zeiss Axio Observer Z1 fluorescent microscope (Carl Zeiss AG, Germany) equipped with ZEN software for image acquisition. Cells were quantified by counting single‐, double‐, or triple‐positive cells across ten randomly selected fields (magnification ×40), using a 0.0625 mm^2^ graticule integrated into the microscope eyepiece. Results were expressed as the number of cells per mm^2^.

### Real‐Time Quantitative PCR (RT‐qPCR) and Western Blot (WB) Analysis

Markers of METTL3, fibrosis (Col‐I, α‐SMA), M1 macrophages (iNOS, IL‐1β, TNF‐α), and M2 macrophages (arginase‐1, CD206, TNF‐β, IL‐10) were assessed using RT‐qPCR and western blot, respectively. For RT‐qPCR, total RNA was extracted from kidney tissues or cell isolates using RNAiso Plus (Trizol) (Takara, Japan). Reverse transcription was performed using 1 µg of total RNA, followed by quantitative PCR on an S1000 Thermal Cycler (Bio‐Rad, Germany) using Rox dye (Invitrogen), FastStart Taq Polymerase (Roche Diagnostics, Mannheim, Germany), and gene‐specific primers with Fam‐Tamra–labeled probes (BioTez, Berlin, Germany). The PCR conditions were initial denaturation at 96 °C for 10 min, followed by 40 cycles of 10 s at 95 °C and 1 min at 60 °C. GAPDH was used as a housekeeping gene, and the relative gene expression was calculated using the ΔΔCt method. For western blot, proteins were extracted using RIPA lysis buffer and quantified by BCA protein assay (Bio‐Rad). Each sample containing 20 µg of protein was separated on 10% SDS‐polyacrylamide gel (Bio‐Rad) and transferred to a polyvinylidene difluoride (PVDF) membrane (Sigma‐Aldrich). Membranes were incubated overnight at 4 °C with primary antibodies targeting α‐SMA, Col‐I, METTL3, and GAPDH. Detection was performed using an Odyssey infrared imaging system (LI‐COR). Protein levels were quantified using ImageJ across three independent experiments, and results were normalized to GAPDH as the internal control.

### *RNA*‐Sequencing Analysis

Total RNA was extracted from BMDMs treated with TGF‐β1 for 72 h or the control group and subjected to RNA‐seq using an Illumina Novaseq platform. Gene set enrichment analysis (GSEA) was performed on normalized RNA‐seq transcript data for functional annotation.

### Quantification of m6A Modification

Total RNA (500 ng) was analyzed using an m6A RNA Methylation Assay Kit (Abcam, Cambridge, MA, USA) following the manufacturer's protocol to quantify global m6A methylation levels. Additionally, the mRNA expression levels of five key m6A regulators (METTL3, METTL14, WTAP, ALKBH5, and FTO) were determined by RT‐qPCR. Total RNA was reverse transcribed using 1 µg of RNA, and gene‐specific primers were designed based on sequences from public databases. GAPDH was used as the housekeeping gene for normalization, and relative expression was calculated using the ΔΔCt method.

### Flow Cytometry

Single cells were harvested from the kidney tissue of mice or patients with chronic renal allograft rejection using collagenase digestion (0.5 mg mL^−1^, 30‐min incubation, Worthington). After isolation, cells were permeabilized and treated with FITC‐conjugated antibodies against F4/80, CD11b, α‐SMA, Col‐I, and METTL3 (eBioscience, San Diego, CA, USA), following the manufacturer's recommended dilutions. Isotype‐matched control antibodies and unstained cells served as negative controls. Flow cytometry was performed using a Cytek Aurora flow cytometer (Cytek Biosciences, USA), and data were analyzed using SpectroFlo software with compensation applied to correct for spectral overlap.

### Statistical Analysis

Results are presented as the mean ± standard error of the mean (SEM). Data were evaluated using a *t*‐test for unpaired samples, with a *p*‐value < 0.05 indicating statistical significance. For multiple comparisons, one‐way ANOVA was used followed by posthoc Tukey correction. All statistical analyses were performed using GraphPad Prism software.

### Ethics Approval

The collection of renal allograft biopsy specimens was approved by the Ethics Committee of Zhejiang University (Ethical Approval No. 2022422) and informed consent was obtained from all participants prior to sample collection. Animal experiments were conducted in accordance with ethical guidelines and were approved by the Animal Experimental Ethics Committee of The First Affiliated Hospital, Zhejiang University School of Medicine (project number 2022646)).

## Conflict of Interest

The authors declare no conflict of interest.

## Author Contributions

Q.Y. and X.Z. contributed equally to this work. Q.Y. performed all experiments, data collection, and analyses, and wrote the manuscript. X.Z. X.Z., and Y.W. involved in animal experiments and histological, and statistical analyses. Q.Z., J.L., L.Z., and J.L. took part in data collection and interpreted the results. D.C., W.C., and J.C. conceived and supervised the whole project. All authors read and approved the final manuscript.

## Data Availability

The data are available from the corresponding author upon reasonable request.
